# O Escore de Selvester QRS é um Preditor de Mortalidade na Insuficiência Cardíaca com Fração de Ejeção Preservada

**DOI:** 10.36660/abc.20230235

**Published:** 2023-09-19

**Authors:** Fatih Sivri, Yahya Kemal Icen, Hasan Koca, Mükremin Coşkun, Mustafa Ardınç, Orshan Deniz, Fatih Necip Arici, Mevlüt Koc, Hasan Güngör

**Affiliations:** 1 Nazilli State Hospital Department of Cardiology Aydin Turquia Nazilli State Hospital – Department of Cardiology, Aydin – Turquia; 2 Adana Health Practice and Research Center Department of Cardiology Adana Turquia Adana Health Practice and Research Center – Department of Cardiology, Adana – Turquia; 3 Adnan Menderes University Department of Cardiology Aydin Turquia Adnan Menderes University – Department of Cardiology, Aydin – Turquia

**Keywords:** Selvester QRS, Mortalidade, Insuficiência Cardíaca, Infarto do Miocárdio, Eletrocardiografia/métodos, Ecocardiografia/métodos, Volume Sistólico

## Abstract

**Fundamento:**

O escore Selvester QRS (S-QRS) em um eletrocardiograma (ECG) de 12 derivações está associado tanto à quantidade de cicatriz miocárdica quanto ao mau prognóstico em pacientes com infarto do miocárdio. Entretanto, seu valor prognóstico na insuficiência cardíaca (IC) com fração de ejeção preservada (ICFEp) é desconhecido.

**Objetivo:**

Este estudo tem como objetivo investigar o valor preditivo do escore S-QRS para mortalidade na ICFEp.

**Métodos:**

359 pacientes foram incluídos retrospectivamente neste estudo. As características eletrocardiográficas, ecocardiográficas e laboratoriais dos pacientes foram registradas. O escore S-QRS simplificado foi medido e registrado. O tempo médio de seguimento dos pacientes foi de 38,1±9,5 meses. A significância estatística foi estabelecida em p < 0,05.

**Resultados:**

Dos 359 pacientes, 270 estavam no grupo sobrevivente e 89 no grupo falecido. Idade, PCR-us, troponina, pro-BNP, diâmetro do átrio esquerdo (AE), índice de volume do AE, duração do QRS, Tpe e escore do S-QRS foram estatisticamente altos no grupo falecido. Na análise de regressão logística multivariada, idade, PCR-us, NT-proBNP, diâmetro do AE, índice de volume do AE, Tpe e escore S-QRS mostraram-se fatores de risco independentes para mortalidade. Na análise da característica operacional do receptor (ROC), o valor de corte do escore S-QRS foi de 5,5, a sensibilidade foi de 80,8% e a especificidade foi de 77,2% (AUC: 0,880, p:0,00). Na análise de Kaplan-Meier, verificou-se que a mortalidade foi maior no grupo com escore S-QRS ≥ 5,5 do que no grupo com escore S-QRS < 5,5. (Long-rank, p:0,00)

**Conclusão:**

Acreditamos que o escore S-QRS pode ser usado como um indicador prognóstico de mortalidade a longo prazo em pacientes com ICFEp.

## Figura Central

**Figure:**
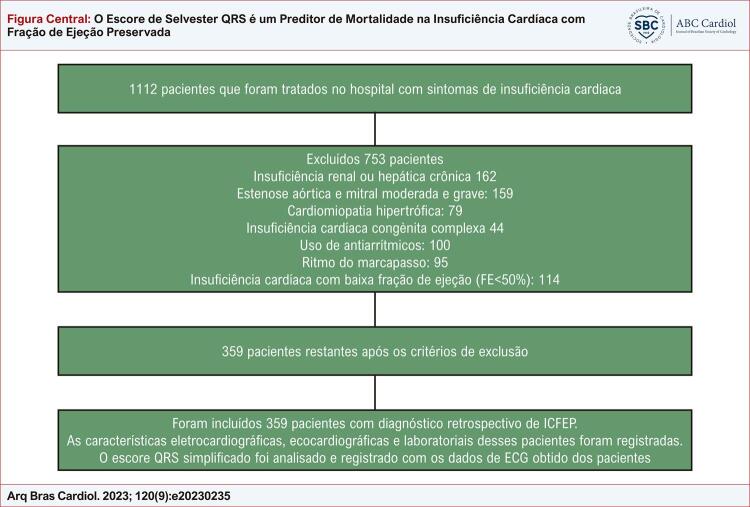
: O Escore de Selvester QRS é um Preditor de Mortalidade na Insuficiência Cardíaca com Fração de Ejeção Preservada

O diagrama mostra a seleção dos grupos de estudo.

## Introdução

A insuficiência cardíaca (IC) é detectada em 1-2% dos adultos. Sua incidência aumenta com a idade. Enquanto é de 1% em indivíduos <55 anos, é de aproximadamente 10% em indivíduos > 70 anos.^1^ De acordo com a última diretriz, a IC é basicamente dividida em 3 classes: IC com fração de ejeção preservada (ICFEp), IC com fração de ejeção leve (ICFEm) e IC com fração de ejeção limítrofe (ICFEl). A ICFEp apresenta-se clinicamente com sintomas de IC e fração de ejeção normal ou quase normal (FE > 50%).^2^ Representa aproximadamente 50% dos pacientes atendidos em hospitais por IC. Em estudos epidemiológicos, < 70% dos pacientes com ICFEp têm mais de 65 anos, e ICFEp é observada em quase todos os pacientes com IC acima de 90 anos de idade.^3,4^

Hipertrofia ventricular esquerda, inflamação sistêmica e miocárdica, dano endotelial microvascular e infarto, estresse oxidativo e fibrose intersticial miocárdica foram observados como fatores fisiopatológicos subjacentes na ICFEp.^5^ Estudos demonstraram que a fibrose intersticial miocárdica é um dos mecanismos fisiopatológicos mais importantes da doença e um indicador prognóstico de longo prazo.^6,7^

Embora dispositivos médicos avançados tenham nos fornecido informações novas e importantes, o eletrocardiograma (ECG) padrão de 12 derivações ainda é o principal método que fornece informações cruciais. Em 1970, Selvester et al.,^8^ desenvolveram um sistema de escore de 31 pontos (QRS) que avaliou a mudança na despolarização ventricular devido à cicatriz miocárdica em um ECG padrão de 12 derivações. Cada escore correspondeu a 3% da massa do músculo ventricular esquerdo.^8^ Em estudos de ressonância magnética cardíaca de cicatrizes miocárdicas, foi detectado que a escore Selvester QRS (S-QRS) se correlaciona altamente com o tamanho da cicatriz.^9^ Em muitos estudos clínicos, um escore S-QRS alto demonstrou fornecer informações sobre o tamanho do infarto que se desenvolve após o infarto do miocárdio com elevação do segmento ST e o prognóstico de longo prazo dos pacientes.^10,11^ Seu valor prognóstico foi relatado em muitos estudos cardiovasculares doenças, como cardiomiopatia não isquêmica, estenose aórtica e cardiomiopatia hipertrófica.^12-14^ No entanto, não há informações sobre sua associação com ICFEp.

Este estudo tem como objetivo investigar o valor preditivo do escore S-QRS para mortalidade na ICFEp.

## Métodos

### População de pacientes

Este estudo retrospectivo incluiu pacientes com ICFEp tratados para sintomas de IC em condições hospitalares em um único centro entre 2018 e 2022 após aprovação do comitê de ética local. Mil e doze pacientes com IC foram examinados e 359 pacientes foram diagnosticados com ICFEp. Os pacientes foram acompanhados por uma média de 38,1±9,5 meses. Esses critérios diagnósticos foram fração de ejeção do ventrículo esquerdo (FEVE) ≥50%, pró-peptídeo natriurético cerebral N-terminal (NT-proBNP) > 125 pg/m, e também um dos dois critérios, (1) hipertrofia ventricular esquerda ou aumento do átrio esquerdo (AE), (2) disfunção diastólica (E/e ≥ 13 e média e’ septal e parede lateral < 9 cm/s ao ecocardiograma Doppler).

Pacientes com insuficiência renal ou hepática crônica, estenose aórtica e mitral moderada e grave, cardiomiopatia hipertrófica, IC congênita complexa, uso de antiarrítmicos, ritmo de marca-passo, IC com baixa fração de ejeção (FE < 50%), síndrome coronariana aguda, câncer, sepse e níveis anormais de eletrólitos séricos foram excluídos do estudo. Além disso, foi assegurado nos pacientes que o traçado do ECG era de boa qualidade, ou seja, sem bloqueio de ramo esquerdo ou direito, sem bloqueio fascicular anterior ou posterior esquerdo, sem hipertrofia ventricular esquerda ou direita, sem síndrome de Wolff-Parkinson-White, sem baixa voltagem ou estimulação ventricular que pudesse interferir na determinação do escore S-QRS. A seleção do grupo de estudo é resumida na ilustração central.

Todos os pacientes foram exaustivamente questionados sobre hipertensão, hiperlipidemia, diabetes mellitus, tabagismo, doença arterial coronariana e acidente vascular cerebral. Os valores hematológicos, bioquímicos e sorológicos foram determinados e registrados a partir do sangue periférico coletado após 12 horas de jejum.

A insuficiência renal crônica foi definida como uma taxa de filtração glomerular inferior a 60 por mais de 3 meses. Um diagnóstico de hipertensão foi aceito se os pacientes estivessem em tratamento anti-hipertensivo ou tivessem uma pressão arterial sistólica superior a 140 mmHg e pressão arterial diastólica de 90 mmHg em pelo menos três medições. Diabetes foi diagnosticado se os pacientes estivessem tomando medicação antidiabética, tivessem pelo menos duas medições de glicemia pós-prandial acima de 126 mg/dl ou tivessem um nível de HbA1c > 6,5. Lipoproteína de baixa densidade (LDL) > 160 mg/dl ou uso de estatinas foi aceito como diagnóstico de hiperlipidemia. Para o diagnóstico de doença arterial coronariana, assumiu-se estenose > 50% em pelo menos uma artéria coronária epicárdica. O estado atual dos pacientes foi determinado e registrado entrando em contato com os controles do hospital e por telefone.

### Avaliação ecocardiográfica

Imagens bidimensionais e coloridas de Doppler nas visualizações paraesternal padrão de eixo longo, eixo curto e apical foram obtidas e analisadas on-line por um ecocardiologista experiente cego para os dados clínicos. O exame ecocardiográfico de todos os pacientes incluídos no estudo foi realizado com um sistema de ultrassom cardíaco iE33 (Phillips Healthcare, Best, Holanda) e um sistema de sonda de 2,5-5 MHz. Todas as medições ecocardiográficas relatadas foram calculadas a partir de três ciclos consecutivos. A função sistólica global do ventrículo direito foi medida como a excursão sistólica do plano anular tricúspide (TAPSE) usando a diferença bidimensional entre as linhas diastólica final e sistólica final (em cm) entre o centro da origem do ventilador do ultrassom e a junção do anel tricúspide lateral do ventrículo direito no corte apical quatro câmaras. As imagens da veia cava inferior (VCI) foram adquiridas na visão subxifoide, e o diâmetro transverso (VCId) foi medido anterior a 2 cm posterior da junção atrial direita da VCI com o modo M no diâmetro máximo durante a expiração. A velocidade de pico da regurgitação tricúspide foi medida e a pressão sistólica da artéria pulmonar foi estimada da seguinte forma: 4 (velocidade de pico do TR) 2 a ecocardiografia com Doppler pulsado para avaliar as velocidades de enchimento diastólico dos ventrículos foi realizada no corte apical de quatro câmaras. Assim, a velocidade máxima do enchimento diastólico inicial (onda E) e a velocidade máxima do enchimento diastólico tardio (onda A) foram registradas. O volume máximo AE foi determinado a partir das visualizações apicais de quatro câmaras e duas câmaras no final da sístole usando o método modificado do disco de Simpson e, em seguida, normalizado para a área de superfície corporal para derivar o índice de volume do AE.

### Avaliação eletrocardiográfica

Os ECGs superficiais de 12 derivações de todos os pacientes (dispositivo Nihon Kohden Cardiofix V modelo ECG-1550K 25mm/s e padrão 1mv/10mm) foram registrados durante a internação inicial e antes do tratamento da IC e avaliados por dois cardiologistas independentes que não conheciam as características dos pacientes. Manualmente, a frequência cardíaca, intervalo PR, intervalos QT e QTc, duração do QRS e escore do S-QRS foram medidos e registrados. O intervalo PR foi medido em milissegundos pelo tempo entre o início da onda P e o início do complexo QRS. A duração do QRS foi medida em milissegundos pelo tempo entre o início da onda Q ou R e o final da onda R ou S. O intervalo QT foi medido em milissegundos pelo tempo entre o início do complexo QRS e o final da onda T. O intervalo QT corrigido foi medido usando a fórmula de Bazett. O intervalo Tpe foi medido do pico da onda T até o final da onda T. O final da onda T foi definido como a interseção da tangente ao declive da onda T e a linha isoelétrica.

### Medição de escore do QRS Selvester

Os ECGs foram pontuados manualmente de acordo com o sistema simplificado de escore de 37 critérios e 29 pontos de Bounous et al.,^15^. Dois cardiologistas experientes calcularam manualmente a escore S-QRS dependendo de um algoritmo relatado anteriormente. Se os dois escores não concordassem, o terceiro cardiologista calculava o escore do S-QRS de forma cega e o finalizava. O sistema de escore é baseado em critérios para 10 das 12 derivações de um ECG padrão de 12 derivações (aVL, aVF, I, II, V1-6). Principalmente, os pontos são dados para a duração da onda Q, amplitudes e duração R e relação R/S ou R/Q.

### Análise estatística

Os pacotes estatísticos IBM SPSS Statistics for Windows (versão 25.0) (NY, EUA) e Amos (versão 24.0) (WA, EUA) foram usados para analisar os dados. O teste de Kolmogorov-Smirnov foi realizado para determinar se os dados eram normalmente distribuídos. Variáveis contínuas são apresentadas como média (desvio padrão) se a variável for parametricamente distribuída. As variáveis foram comparadas por meio de testes t independentes. Variáveis categóricas são apresentadas como números e porcentagens. O teste do qui-quadrado e o teste exato de Fisher foram realizados para comparar as variáveis categóricas. Um valor de p < 0,05 foi considerado estatisticamente significativo. As variáveis para as quais o p-valor não ajustado no modelo de regressão logística foi < 0,05 foram identificadas como potenciais marcadores de risco e incluídas no modelo multivariado completo. Análises multivariadas de regressão logística com eliminação retrógrada foram realizadas por meio de teste de razão de verossimilhança para eliminação de variáveis. A curva característica de operação do receptor (ROC) foi usada para determinar a sensibilidade e especificidade do escore S-QRS e o valor de corte ideal para prever a mortalidade. As curvas de sobrevida foram estimadas pelo método de Kaplan-Meier. As taxas livres de eventos cardíacos foram comparadas entre os grupos por meio do teste de log-rank.

## Resultados

Dos 359 pacientes, 270 pertenciam ao grupo sobrevivente e 89 ao grupo falecido. Quando comparados os dados demográficos, a idade foi estatisticamente maior no grupo de falecidos. Nenhuma diferença foi encontrada entre os grupos quando os históricos médicos e tratamentos do paciente foram comparados (Tabela 1).

**Tabela 1:** – Comparação de dados demográficos, medicamentos e histórico médico dos pacientes

	Vivos (n=270)	Falecidos (n=89)	p
**Dados demográficos**			
Anos de idade)	68,8±12,0	74,3±12,04	<0,001
Sexo masculino, n, (%)	75 (27,7)	31 (34,8)	0,129
IMC (kg/m^2^)	30,5±6,4	29,9±6,33	0,545
**Histórico médico**			
Tabagismo, n (%)	65(24,0)	30 (33,7)	0,320
DM, n (%)	155(57,4)	54 (60,6)	0,450
HT, n (%)	200(74)	62 (69,6)	0,157
HPL, n (%)	52(19,2)	20 (22,4)	0,582
AVC, n (%)	12 (4)	4 (4,1)	0,321
DAC, n (%)	112(41,4)	40 (44,9)	0,741
**Uso de medicamentos**			
ECA (n, %)	121 (44,8)	44 (49,4)	0,520
BRA (n, %)	88 (32,5)	30 (33,7)	0,410
Β bloqueador (n, %)	63 (23,3)	21 (23,5)	0,321
Furosemida (n, %)	267 (98,8)	87 (97,7)	0,254
Espironolactona (n, %)	85 (31,4)	24 (26,9)	0,253
Anticoagulante (n, %)	12 (4)	3 (3)	0,512
Digoxina (n, %)	6 (2)	5 (5)	0,254
ASA (n, %)	33 (12.3)	18 (20)	0,355

IMC: índice de massa corporal; DAC: doença arterial coronariana; DPOC: doença pulmonar obstrutiva crônica; DM: diabetes melito; HT: hipertensão; HPL: hiperlipidemia; AVC: acidente vascular cerebral; ECA: enzima conversora de angiotensina; BRA: bloqueadores do receptor de angiotensina; ASA: ácido acetilsalicílico.

Quando os dados laboratoriais foram comparados, os valores de PCR-us e NT-proBNP foram estatisticamente maiores no grupo falecido (Tabela 2).

**Tabela 2:** – Comparação dos achados laboratoriais do paciente

	Vivos n=270	Falecidos n=89	p
Glicose (mg/dl)	135,2 ± 75,5	132,1 ± 65,8	0,889
GB (uL)	9,65 ± 3,45	11,12 ± 4,34	0,063
Hb (mg/dl)	13,1 ± 1,22	12,1 ± 1,23	0,172
BUN (mg/dL)	54,2 ± 21,2	53,0 ± 24,2	0,123
Cr (mg/dL)	1,22 ± 0,45	1,21 ± 0,54	0,123
Na (mmol/L)	137,5 ± 4,2	136,1 ± 5,11	0,351
K (mmol/L)	4,41 ± 0,66	4,72 ± 0,22	0,565
Gfr (mL/min/m^2^)	66,4 ± 25,2	58,6 ± 13,59	0,340
Ácido úrico (mg/dL)	7,5 ± 2,4	7,6 ± 2,81	0,584
Proteína total (g/dL)	7,54 ± 1,11	6,54 ± 1,22	0,458
Albumina (g/dL)	3,4 ± 0,28	3,45 ± 1,1	0,254
Cálcio (mg/dL)	8,44 ± 0,556	8,91 ± 0,457	0,234
PCR-us (mg/L)	2,1 ± 0,45	4,1 ± 1,2	0,01
NT-proBNP (pg/ml)	3520 ± 1225	4500 ± 1450	0,01
TnT-us (pg/L)	1,2 ± 0,7	1,4 ± 0,9	0,121

GB: glóbulos brancos; Hb: hemoglobina; BUN: nitrogênio ureico no sangue; Cr: creatinina; Na: sódio; K: potássio; Gfr: taxa de filtração glomerular; PCR-us: proteína C reativa de alta sensibilidade; NT-proBNP: N-terminal peptídeo natriurético cerebral; TnT-us: troponina T altamente sensível.

Ao comparar as características eletrocardiográficas e ecocardiográficas, diâmetro do AE, índice de volume do AE, duração do QRS, Tpe e escore do S-QRS foram estatisticamente maiores no grupo falecido (Tabela 3).

**Tabela 3:** – Comparação dos achados ecocardiográficos e eletrocardiográficos dos pacientes

	Vivos n=270	Falecidos n=89	p
**Achados ecocardíográficos**			
FE (%)	54,5±5,22	54,5±4,78	0,356
DDVE (mm)	46,5 ± 3,1	47,0 ± 3,2	0,198
DSVE (mm)	35,4 ± 1,8	35,5 ±1,8	0,589
EPPVE (mm)	10,4 ± 1,4	10,1 ± 1,3	0,131
SVI (mm)	10,7 ± 2,0	11,3 ± 2,4	0,126
DAE (mm)	44,6±4,1	45,9±3,7	0,001
IVAE (ml/m^2^)	28,4±9,1	41,2±7,1	0,001
Velocidade E (cm/s)	89,8±23,7	92,2±20,1	0,256
Velocidade A (cm/s)	61,1±21,3	62,7±21,8	0,356
Velocidade S (cm/s)	7,2±1,98	7,38±1,92	0,561
Velocidade e' (cm/s)	7,022±1,82	7,01±2,02	0,784
Velocidade a' (cm/s)	4,16±1,74	3,83±1,63	0,231
E/e'	13,4±5,1	13,9±4,7	0,456
PSAP (mmhg)	33,2±8,6	32,2±7,9	0,354
TAPSE, (cm)	1,7±0,35	1,7±0,51	0,259
Diâmetro da VCI (mm)	24±7,2	23±4,7	0,125
**Achados eletrocardiográficos**			
QRS (msn)	87,7±18,2	94,8±25,6	0,002
P duração (ms)	90,3±6,8	89,1±6,07	0,023
Intervalo PR (ms)	160,7±27,2	161,6±31,2	0,541
QT (ms)	388,1±53,7	384,6±61,08	0,154
QTC (ms)	440,4±37,2	447,87±46,93	0,586
TPe (ms)	72,74±17,7	86,12±17,9	0,000
Escore do QRS de Selvester	4,20±1,71	7,213±1,932	0,000

FE: fração de ejeção; DDVE: diâmetro diastólico final do ventrículo esquerdo; DSVE: diâmetro sistólico final do ventrículo esquerdo; EPPVE: espessura da parede posterior do ventrículo esquerdo; SVI: septo interventricular; DAE: diâmetro do átrio esquerdo; IVAE: índice de volume do átrio esquerdo; PSAP: pressão sistólica da artéria pulmonar; Tpe: T pico a fim; TAPSE: excursão sistólica do plano anular tricúspide; VCI: veia cava inferior.

Na análise de regressão logística multivariada, idade, PCR-us, NT-proBNP, diâmetro do AE, índice de volume do AE, Tpe e escore S-QRS mostraram-se fatores de risco independentes para mortalidade (Tabela 4).

**Tabela 4:** – Preditores independentes de mortalidade em pacientes com ICFEp

	OR	IC de 95%	p	OR	%95 IC	p
Idade	1.059	1.012-1.109	0,013	1.022	1.012-1.035	0,001
Duração do QRS	1.010	0,983-1,037	0,476			
DAE	1.302	1.138-1.490	0,001	1.220	1.110-1.350	0,002
Duração da onda P	1.651	1.120-1.235	0,005	1.33	1.240-1.550	0,03
Tpe	1.053	1.022-1.084	0,001	1.131	1.088-1.175	0,000
Escore do QRS de Selvester	2.446	1.783-3.555	0,001	1.588	1.352-1.755	0,000
PCR-us (mg/L)	1.655	1.256-2.122	0,000	1.436	1.115-1.848	0,005
NT-proBNP (pg/ml)	1.211	1.108-1.324	0,000	1.431	1.306-1.696	0,001
TnT-us (pg/L)	1.004	0,989-1,009	0,375			
IVAE	1.056	1.024-1.078	0,001	1.035	1.022-1.055	0,005

DAE: diâmetro do átrio esquerdo; ICFEp: fração de ejeção preservada por insuficiência cardíaca; Tpe: T pico a fim; IVAE: índice de volume atrial esquerdo; PCR-us: proteína c-reativa de alta sensibilidade; NT-proBNP: peptídeo natriurético tipo B N-terminal; TnT-us: troponina T altamente sensível.

**Figura 1:**
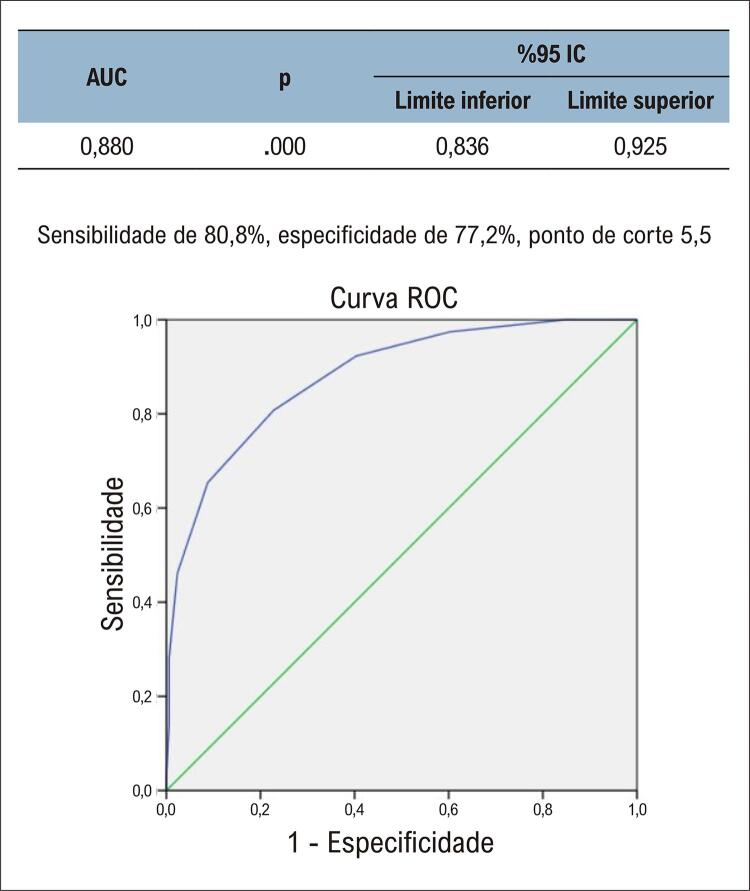
– Análise da curva ROC do escore Selvester QRS. AUC: área sob a curva; IC: intervalo de confiança.

**Figura 2:**
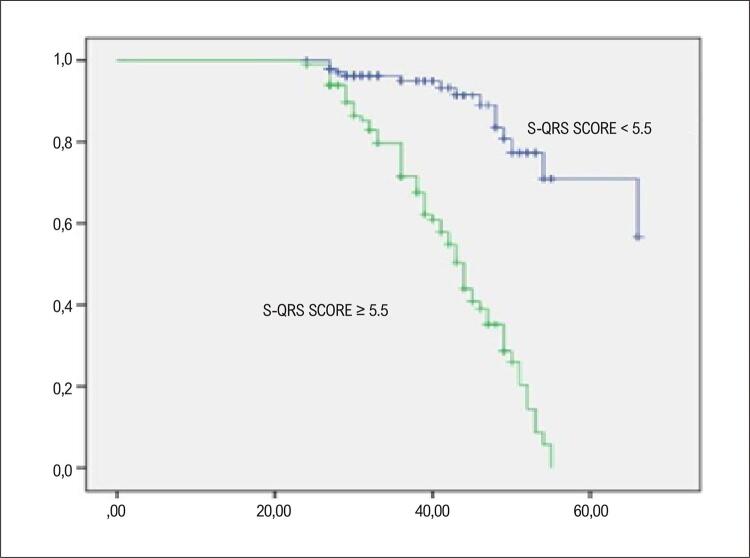
– Análise Kaplan Meier do escore Selvester QRS. A taxa de eventos cardíacos foi significativamente maior no grupo de escore alto (HS) (linha verde) do que no grupo de escore baixo (LS) (linha azul).

Na análise ROC, o valor de corte do escore S-QRS foi 5,5, com sensibilidade de 80,8% e especificidade de 77,2% (AUC: 0,880) (Figura 1). Segundo a análise de Kaplan-Meier, a mortalidade foi maior no grupo com escore S-QRS ≥ 5,5 do que no grupo com escore S-QRS < 5,5. (Long-rank, p:0,00) (Figura 2).

## Discussão

Nosso estudo foi o primeiro a investigar o valor preditivo do escore S-QRS para mortalidade na ICFEp. A análise ROC apresentou sensibilidade e especificidade de 80,8% e 77,2%, respectivamente. Ao final deste estudo, o escore S-QRS mostrou-se um fator de risco independente para mortalidade em longo prazo em pacientes com ICFEp.

Semelhante a estudos anteriores, idade, PCR-us, NT-proBNP, diâmetro do AE, índice de volume do AE e Tpe foram fatores de risco independentes para mortalidade em pacientes com ICFEp. Em estudos clínicos, mortalidade por todas as causas, mortalidade por doença cardiovascular, IC e hospitalização foram observadas com muito mais frequência em idosos do que em jovens.^16,17^ Tromp et al.,^18^ determinaram que a taxa de mortalidade era 6,9 vezes maior e a taxa de hospitalização era 16,9 vezes maior nos maiores de 85 anos do que nos menores de 55 anos.^18^

Em muitos estudos, a PCR-us demonstrou ser um indicador prognóstico muito importante de mortalidade por inflamação e fibrose na fisiopatologia da ICFEp.^19,20^L. Koller et al.,^21^ relataram que a mortalidade por todas as causas aumentou 1,2 vezes e a mortalidade cardiovascular aumentou 1,32 vezes em pacientes com PCR-us elevada, resultando em um acompanhamento médio de 9,7 anos em 459 pacientes com ICFEp.^21^ Foi relatado que o NT-proBNP liberou durante o aumento do estresse da parede miocárdica devido a um ventrículo esquerdo hipertrófico e pequeno, que é o aspecto característico da ICFEp, tem poder preditivo para morbidade e mortalidade a longo prazo em pacientes com ICFEp, tanto em termos de níveis basais quanto de alterações nesses níveis.^22^ Embora se saiba que o valor preditivo dos peptídeos natriuréticos é menor em pacientes com ICFEp do que em pacientes com IC com fração de ejeção baixa, eles mostraram ter o mesmo valor preditivo em ambos os grupos de IC no estudo de van Veldhuisen et al.^23^

Tpe é um marcador de ECG que tem recebido muita atenção nos últimos anos. Em muitos estudos, a dispersão transmural foi aceita como um indicador de anormalidades da repolarização e demonstrou estar associada a arritmias ventriculares e morte súbita.^24^ Estudos em pacientes com ICFEp concluíram que o Tpe é um importante marcador prognóstico, proporcional à gravidade da doença e um fator de risco independente para mortalidade.^25^

A medida do AE é um parâmetro simples, reprodutível e comumente utilizado na prática clínica e em pesquisas. Em estudos realizados em pacientes com ICFEp, o aumento do diâmetro e do índice de volume do AE são considerados indicadores prognósticos para muitas complicações, como fibrilação atrial, hipertensão pulmonar e mortalidade cardiovascular.^26^ Rossi et al.,^27^ apontaram em seu estudo prospectivo que o aumento do diâmetro do AE aumentou a mortalidade em 1,72 vezes.^27^ O estudo de Pate et al.,^28^ descobriram que a mortalidade aumentou 0,9% a cada milímetro de aumento no índice de volume do AE.^28^

O escore S-QRS fornece informações sobre o tamanho e a localização das cicatrizes miocárdicas, examinando as alterações morfológicas do QRS que ocorrem devido a alterações da despolarização ventricular resultantes da fibrose miocárdica.^29^ Muitas autópsias e estudos de ressonância magnética encontraram um alto grau de correlação entre o S-QRS Escore QRS e tamanho da cicatriz.^30-32^ O estudo prospectivo de Liu et al.,^33^ mostraram que a mortalidade cardiovascular aumentou 1,46 vezes em pacientes com escores S-QRS elevados. Isso resultou de um acompanhamento de 2 anos de 289 pacientes após infarto do miocárdio com elevação do segmento ST em comparação com pacientes sem elevação do segmento ST. No estudo de Bignoto et al.,^13^ 228 pacientes submetidos à substituição valvar transcateter para estenose valvar aórtica foram acompanhados por 36,2 ± 21,2 meses e apresentaram uma taxa de mortalidade cardiovascular 1,59 vezes maior em pacientes com altos escores de S-QRS.^13^ No estudo de Hirawi et al.,^12^ uma taxa 1,32 vezes maior de eventos cardíacos fatais foi observada em pacientes com altos escores S-QRS após seguimento médio de 4,5 ± 3,2 anos em 91 pacientes com cardiomiopatia não isquêmica. Além disso, foi encontrada uma alta correlação com o escore S-QRS da fração de colágeno medida por biópsia endomiocárdica.^12^ Uyarel et al.,^34^ demonstraram o desenvolvimento de um fenômeno de no-reflow e alta mortalidade em 30 dias após infarto do miocárdio com elevação do segmento ST em pacientes com alto escore S-QRS.^34^ No estudo de Arisoy et al.,^35^ foi demonstrado que um alto escore S-QRS é um fator de risco independente para taquicardia ventricular e/ou fibrilação ventricular em pacientes com cardiomiopatia não isquêmica.^35^ No estudo de Chen et al.,^36^ que comparou a ressonância magnética (RM) cardíaca e o escore S-QRS em pacientes com cardiomiopatia hipertrófica , notou-se que o escore S-QRS indicou a presença e o tamanho da cicatriz com a mesma precisão da RM cardíaca.^36^ Netsi et al.,^37^ por outro lado, mostraram que o escore S-QRS antes da implantação da terapia de ressincronização cardíaca (TRC) é um dos mais importantes indicadores de resposta ao tratamento TRC.^37^ Muitos estudos revelaram que a fibrose cardíaca é um dos mecanismos fisiopatológicos mais importantes em pacientes com ICFEp. O estudo da autópsia de Mohammed et al.,^38^ provou que doença arterial coronariana epicárdica, infartos microvasculares e cicatrizes macroscópicas e microscópicas eram mais prevalentes em pacientes com ICFEp em comparação com o grupo controle.^38^ No estudo de ressonância magnética cardíaca de Garg et al.,^39^ o tamanho da fibrose foi determinado como um risco independente indicador de mortalidade em pacientes com ICFEp.^39^ Cho et al.,^40^ relataram que o tamanho da fibrose foi um fator de risco independente para o desenvolvimento de arritmias ventriculares em pacientes com ICFEp.^40^ Em um estudo de ressonância magnética cardíaca de Kanagala et al.,^41^ em pacientes com ICFEp, o tamanho da fibrose foi considerado um fator de risco independente para remodelamento biventricular e do AE, bem como hospitalização e mortalidade.^41^ O escore S-QRS é um sistema de pontuação simples, barato e amplamente aceito que mede o tamanho da cicatriz ventricular e é obtido com um ECG padrão de 12 derivações. Em nosso estudo, o escore S-QRS foi considerado um fator de risco independente para mortalidade em pacientes com ICFEp.

### Limitações

Este estudo tem muitas limitações. Primeiro, o número de pacientes incluídos no estudo é pequeno e os registros de ECG não foram examinados durante os exames de rotina. Além disso, valores como PCR e troponina, que estão associados a dano miocárdico subclínico, não foram acompanhados seriadamente. A ressonância magnética cardíaca, padrão-ouro para medir a fibrose ventricular, não foi realizada.

## Conclusão

O escore S-QRS medido pelo ECG padrão de 12 derivações foi considerado um fator de risco independente para mortalidade em pacientes com ICFEp. Portanto, fornece informações sobre a mortalidade do paciente mesmo na ausência de acesso à RM cardíaca e quando outros parâmetros de ECG são normais. Recomendamos que o escore S-QRS não seja negligenciado na avaliação de pacientes de alto risco.
