# Supplementary material for: O Escore de Selvester QRS é um Preditor de Mortalidade na Insuficiência Cardíaca com Fração de Ejeção Preservada
Source: Arq Bras Cardiol. 2023 Sep 19;120(9):e20230235. [Article in Portuguese] doi: 10.36660/abc.20230235 (PMC10519357; doi:10.36660/abc.20230235)
Supplement: Translation

# Translation: Selvester QRS Score is a Predictor of Mortality in Heart Failure with Preserved Ejection Fraction

**Authors:** Fatih Sivri, Yahya Kemal Icen, Hasan Koca, Mükremin Coşkun, Mustafa Ardınç, Orshan Deniz, Fatih Necip Arici, Mevlüt Koc, Hasan Güngör

**Affiliations:** 1 Turkey Nazilli State Hospital – Department of Cardiology Aydin – Turkey; 2 Turkey Adana Health Practice and Research Center – Department of Cardiology, Adana – Turkey; 3 Turkey Adnan Menderes University – Department of Cardiology, Aydin – Turkey

**Keywords:** Selvester QRS, Mortality, Heart Failure, Myocardial Infarction, Electrocardiography/methods, Echocardiography/methods, Stroke Volume

## Abstract

**Background:**

The Selvester QRS (S-QRS) score on a 12-lead electrocardiogram (ECG) is associated with both the amount of myocardial scar and poor prognosis in myocardial infarction patients. However, its prognostic value in heart failure (HF) with preserved ejection fraction (HFpEF) is unknown.

**Objective:**

This study aims to investigate the predictive value of the S-QRS score for mortality in HFpEF.

**Methods:**

359 patients were retrospectively enrolled in this study. Electrocardiographic, echocardiographic, and laboratory features of the patients were recorded. The simplified S-QRS score was measured and recorded. The mean follow-up time of the patients was 38.1±9.5 months. Statistical significance was set at p < 0.05.

**Results:**

Of 359 patients, 270 were in the survivor group, and 89 were in the deceased group. Age, Hs-CRP, troponin, pro-BNP, left atrial (LA) diameter, LA volume index, QRS duration, Tpe, and S-QRS score were statistically high in the deceased group. In multivariate logistic regression analysis, age, Hs-CRP, NT-proBNP, LA diameter, LA volume index, Tpe, and S-QRS score were shown to be independent risk factors for mortality. In the receiver-operating characteristic (ROC) analysis, the cut-off value of the S-QRS score was 5.5, the sensitivity was 80.8%, and the specificity was 77.2% (AUC:0.880, p:0.00). In Kaplan-Meier analysis, it was found that mortality was higher in the group with S-QRS score ≥ 5.5 than in the group with S-QRS score < 5.5. (Long-rank, p:0.00)

**Conclusions:**

We think that the S-QRS score can be used as a prognostic indicator of long-term mortality in patients with HFpEF.

## Introduction

Heart failure (HF) is detected in 1-2% of adults. Its incidence increases with age. While it is 1% in individuals aged < 55 years, it is approximately 10% in individuals aged > 70 years.^1^ According to the latest guideline, HF is basically divided into 3 classes: HF with preserved ejection fraction (HFpEF), HF with mild-range ejection fraction (HFmrEF), and HF with low ejection fraction (HFlEF). Heart failure with preserved ejection fraction presents clinically with symptoms of HF and a normal or near-normal ejection fraction (EF > 50%).^2^ It accounts for approximately 50% of patients treated in hospitals for HF. In epidemiological studies, < 70% of HFpEF patients are over 65, and HFpEF is observed in almost all patients with HF over 90 years of age.^3,4^

Left ventricular hypertrophy, systemic and myocardial inflammation, microvascular endothelial damage and infarction, oxidative stress, and myocardial interstitial fibrosis have been observed as underlying pathophysiological factors in HFpEF.^5^ Studies have shown that myocardial interstitial fibrosis is both one of the most important pathophysiological mechanisms of the disease and a long-term prognostic indicator.^6,7^

Although advanced medical devices have provided us with new and important information, the standard 12-lead electrocardiogram (ECG) is still the main method that provides crucial information. In 1970, Selvester et al.^8^ developed a 31-point scoring system (QRS) that assessed the change in ventricular depolarization due to myocardial scar on a standard 12-lead ECG. Each score corresponded to 3% of left ventricular muscle mass.^8^ In cardiac magnetic resonance studies of myocardial scars, the Selvester QRS (S-QRS) score has been detected to correlate highly with scar size.^9^ In many clinical studies, a high S-QRS score has been shown to provide information about the infarct size that develops after myocardial infarction with ST elevation and the long-term prognosis of patients.^10,11^ Its prognostic value has been reported in many cardiovascular diseases, such as non-ischemic cardiomyopathy, aortic stenosis, and hypertrophic cardiomyopathy.^12-14^ However, there is no information on its association with HFpEF.

This study aims to investigate the predictive value of the S-QRS score for mortality in HFpEF.

## Methods

### Patient population

This retrospective study included patients with HFpEF treated for HF symptoms under hospital conditions at a single center between 2018 and 2022 after approval by the local ethics committee. One thousand one hundred twelve patients with heart failure were examined, and 359 patients were diagnosed with HFpEF. Patients were followed up for a mean of 38.1±9.5 months. These diagnostic criteria were left ventricular ejection fraction (LVEF) ≥50%, N-terminal pro-brain natriuretic peptide (NT-proBNP) > 125 pg/m, and also one of two criteria, (1) left ventricular hypertrophy or enlargement of the left atrium, (2) diastolic dysfunction (E/e ≥ 13 and a mean e’ septal and lateral wall < 9 cm/s on Doppler echocardiography).

Patients with chronic renal or hepatic failure, moderate and severe aortic and mitral stenosis, hypertrophic cardiomyopathy, complex congenital HF, use of anti-arrhythmic drugs, pacemaker rhythm, HF with low ejection fraction (EF < 50%), acute coronary syndrome, cancer, sepsis, and abnormal serum electrolyte levels were excluded from the study. In addition, it was ensured in patients that the ECG trace was of good quality, i.e., with no left or right bundle branch block, no left anterior or posterior fascicular block, no left or right ventricular hypertrophy, no Wolff-Parkinson-White syndrome, no low voltage or ventricular pacing that could interfere with the determination of the S-QRS score. The selection of the study group is summarized in the central illustration.

All patients were thoroughly questioned about hypertension, hyperlipidemia, diabetes mellitus, tobacco smoking, coronary artery disease, and stroke. Hematological, biochemical, and serological values were determined and recorded from peripheral blood drawn after 12 hours of fasting.

Chronic renal failure was defined as a glomerular filtration rate of less than 60 for over 3 months. A diagnosis of hypertension was accepted if patients were taking antihypertensive treatment or had a systolic blood pressure greater than 140 mmHg and diastolic blood pressure of 90 mmHg on at least three measurements. Diabetes was diagnosed if patients were taking antidiabetic medication, had at least two postprandial blood glucose measurements above 126 mg/dl, or had an HbA1c level > 6.5. Low-density lipoprotein (LDL) > 160 mg/dl or taking statins was accepted as a diagnosis of hyperlipidemia. For the diagnosis of coronary artery disease, stenosis > 50% in at least one epicardial coronary artery was assumed. The current status of patients was determined and recorded by contacting hospital controls and by telephone.

### Echocardiographic evaluation

Two-dimensional and color Doppler images in the standard parasternal long-axis, short-axis, and apical views were obtained and analyzed online by an experienced echocardiologist blinded to the clinical data. Echocardiographic examination of all patients included in the study was performed with an iE33 cardiac ultrasound system (Phillips Healthcare, Best, The Netherlands) and a 2.5-5-MHz probe system. All reported echocardiographic measurements were averaged from three consecutive cycles. Global right ventricular systolic function was measured as the tricuspid annular plane systolic excursion using the two-dimensional difference between the end-diastolic and end-systolic lines (in cm) between the center of the ultrasound fan origin and the junction of the right ventricular lateral tricuspid annulus in the apical four-chamber view. Images of the inferior vena cava (IVC) were acquired in the subxiphoid view, and transverse diameter (IVCd) was measured anterior to posterior 2 cm from the IVC right atrial junction with M-mode at maximal diameter during expiration. Peak velocity of tricuspid regurgitation was measured, and pulmonary artery systolic pressure was estimated as follows: 4 (TR peak velocity) 2 Pulsed Doppler echocardiography to assess diastolic filling velocities of the ventricles was performed in the apical four-chamber view. Thus, peak velocity of early diastolic filling (E-wave) and peak velocity of late diastolic filling (A-wave) were recorded. Maximal volume LA was determined from the apical four-chamber and two-chamber views at the end of systole using the modified Simpson disk method and then normalized to body surface area to derive left atrium volume index.

### Electrocardiographic evaluation

The superficial 12-lead ECGs of all patients (Nıhon Kohden Cardiofix V model ECG-1550K device 25mm/s and standard 1mv/10mm) were recorded during the initial hospitalization and before treatment of HF and evaluated by two independent cardiologists who did not know the patients’ characteristics. Manually, heart rate, P-R interval, QT and QTc intervals, QRS duration, and S-QRS score were measured and recorded. The P-R interval was measured in milliseconds by the time between the onset of the P-wave and the onset of the QRS complex. The QRS duration was measured in milliseconds by the time between the onset of the Q- or R-wave and the end of the R- or S-wave. The QT interval was measured in milliseconds by the time between the onset of the QRS complex and the end of the T-wave. The corrected QT interval was measured using the Bazett formula. The Tpe interval was measured from the peak of the T-wave to the end of the T-wave. The end of the T-wave was defined as the tangent’s intersection to the T-wave’s downslope and the isoelectric line.

### Selvester QRS score measurement

ECGs were manually scored according to the simplified 37-criterion 29-point scoring system of Bounous et al.,^15^. Two experienced cardiologists manually calculated the S-QRS score depending on an algorithm previously reported. If the two scores did not agree, the third cardiologist calculated the S-QRS score in a blinded fashion and finalized it. The scoring system is based on criteria for 10 of the 12 leads of a standard 12-lead ECG (aVL, aVF, I, II, V1-6). Mainly, points are given for Q-wave duration, R amplitudes and duration, and R/S or R/Q ratio.

### Statistical analysis

The statistical packages IBM SPSS Statistics for Windows (version 25.0) (NY, USA) and Amos (version 24.0) (WA, USA) were used to analyze the data. The Kolmogorov-Smirnov test was performed to determine if the data were normally distributed. Continuous variables are presented as mean (standard deviation) if the variable is parametrically distributed. Variables were compared using independent t-tests. Categorical variables are presented as numbers and percentages. The chi-square and Fisher’s exact tests were performed to compare categorical variables. A p-value < 0.05 was considered statistically significant. Variables for which the unadjusted p-value in the logistic regression model was < 0.05 were identified as potential risk markers and included in the full multivariate model. Multivariate logistic regression analyses with backward elimination were performed using a likelihood ratio test to eliminate variables. The receiver-operating characteristic (ROC) curve was used to determine the sensitivity and specificity of the S-QRS score and the optimal cut-off value for predicting mortality. Survival curves were estimated by the Kaplan-Meier method. The cardiac event-free rates were compared between groups using the log-rank test.

## Results

Of 359 patients, 270 belonged to the survivor group and 89 to the deceased group. When demographic data were compared, age was statistically higher in the deceased group. No difference was found between the groups when the patient’s medical histories and treatments were compared (Table 1).

**Table 1:** – Comparison of patients’ demographic, medications, and medical history

	Living (n=270)	Deceased (n=89)	p
**Demographics Features**			
Age (years)	68.8±12.0	74.3±12.04	<0.001
Male gender,n,(%)	75 (27.7)	31 (34.8)	0.129
BMI (kg/m2)	30.5±6.4	29.9±6.33	0.545
**Medical History**			
Smoking, n (%)	65(24.0)	30 (33.7)	0.320
DM, n (%)	155(57.4)	54 (60.6)	0.450
HT, n (%)	200(74)	62 (69.6)	0.157
HPL, n (%)	52(19.2)	20 (22.4)	0.582
Stroke, n (%)	12 (4)	4 (4.1)	0.321
CAD, n (%)	112(41.4)	40 (44.9)	0.741
**Medication use**			
ACE (n, %)	121 (44.8)	44 (49.4)	0.520
ARB (n, %)	88 (32.5)	30 (33.7)	0.410
Β blocker (n, %)	63 (23.3)	21 (23.5)	0.321
Furosemid (n, %)	267 (98.8)	87 (97.7)	0.254
Spironolactone (n, %)	85 (31.4)	24 (26.9)	0.253
Anticoagulant (n, %)	12 (4)	3 (3)	0.512
Digoksin (n, %)	6 (2)	5 (5)	0.254
ASA (n, %)	33 (12.3)	18 (20)	0.355

BMI: body mass index; CAD: coronary artery disease; COPD: chronic obstructive pulmonary disease; DM: diabetes mellitus; HT: hypertension; HPL: hyperlipidemia; ACE: angiotensin-converting enzyme; ARB: angiotensin receptor blockers; ASA: acetyl salicylic acid.

When laboratory data were compared, Hs-CRP and NT-proBNP values were statistically higher in the deceased group (Table 2).

**Table 2:** – Comparison of patient’s laboratory findings

	Living n=270	Deceased n=89	p
Glucose (mg/dl)	135.2 ± 75.5	132.1 ± 65.8	0.889
WBC (uL)	9.65 ± 3.45	11.12 ± 4.34	0.063
Hb (mg/dl)	13.1 ± 1.22	12.1 ± 1.23	0.172
BUN (mg/dL )	54.2 ± 21.2	53.0 ± 24.2	0.123
Cr (mg/dL)	1.22 ± 0.45	1.21 ± 0.54	0.123
Na (mmol/L)	137.5 ± 4.2	136.1 ± 5.11	0.351
K (mmol/L)	4.41 ± 0.66	4.72 ± 0.22	0.565
Gfr (mL/min/m^2^)	66.4 ± 25.2	58.6 ± 13.59	0.340
Uricacid (mg/dL)	7.5 ± 2.4	7.6 ± 2.81	0.584
Total protein (g/dL)	7.54 ± 1.11	6.54 ± 1.22	0.458
Albumin (g/dL)	3.4 ± 0.28	3.45 ± 1.1	0.254
Calcium (mg/dL)	8.44 ± 0.556	8.91 ± 0.457	0.234
Hs-CRP (mg/L)	2.1 ± 0.45	4.1 ± 1.2	0.01
NT-proBNP (pg/ml)	3520 ± 1225	4500 ± 1450	0.01
Hs-TnT (pg/L)	1.2 ± 0.7	1.4 ± 0.9	0.121

WBC: white blood cells; Hb: hemoglobin; BUN: blood urea nitrogen; Cr: creatinine; Na: sodium; K: potassium; Gfr: glomerular filtration rate; Hs-CRP: high sensitive C reactive protein; NT-proBNP: N-terminal brain natriuretic peptide; Hs-TnT: high sensitive troponin T.

When comparing electrocardiographic and echocardiographic characteristics, LA diameter, LA volume index, QRS duration, Tpe, and S-QRS score were statistically higher in the deceased group (Table 3).

**Table 3:** – Comparison of patients’ echocardiographic and electrocardiographic findings

	Living n=270	Deceased n=89	p
**Echocardiographic findings**			
EF (%)	54.5±5.22	54.5±4.78	0.356
LVDD (mm)	46.5 ± 3.1	47.0 ± 3.2	0.198
LVDS (mm)	35.4 ± 1.8	35.5 ±1.8	0.589
LVPWT (mm)	10.4 ± 1.4	10.1 ± 1.3	0.131
IVSD (mm)	10.7 ± 2.0	11.3 ± 2.4	0.126
LAD (mm)	44.6±4.1	45.9±3.7	0.001
LAVI (ml/m^2^)	28.4±9.1	41.2±7.1	0.001
E velocity (cm/s)	89.8±23.7	92.2±20.1	0.256
A velocity (cm/s)	61.1±21.3	62.7±21.8	0.356
S velocity (cm/s)	7.2±1.98	7.38±1.92	0.561
e' velocity (cm/s)	7.022±1.82	7.01±2.02	0.784
a'velocity (cm/s)	4.16±1.74	3.83±1.63	0.231
E/e'	13.4±5.1	13.9±4.7	0.456
PAPs (mmhg)	33.2±8.6	32.2±7.9	0.354
TAPSE, (cm)	1.7±0.35	1.7±0.51	0.259
IVC diameter (mm)	24±7.2	23±4.7	0.125
**Electrocardiographic findings**			
QRS (msn)	87.7±18.2	94.8±25.6	0.002
P duration (ms)	90.3±6.8	89.1±6.07	0.023
PR interval (ms)	160.7±27.2	161.6±31.2	0.541
QT (ms)	388.1±53.7	384.6±61.08	0.154
QTC (ms)	440.4±37.2	447.87±46.93	0.586
TPe (ms)	72.74±17.7	86.12±17.9	0,000
Selvester QRS score	4.20±1.71	7.213±1.932	0,000

EF: ejection fraction; LVDD: left ventricular end diastolic diameter; LVSD: left ventricular end systolic diameter; LVPWT: left ventricular posterior wall thickness; IVSD: interventricular septum; LAD: left atrium diameter; LAVI: left atrium volume index; PAPs: systolic pulmonary artery pressure; Tpe: T peak to end; TAPSE: tricuspid annular plane systolic excursion; IVC: inferior vena cava

In multivariate logistic regression analysis, age, Hs-CRP, NT-proBNP, LA diameter, LA volume index, Tpe, and S-QRS score were shown to be independent risk factors for mortality (Table 4).

**Table 4:** – Independent predictors for mortality in patients with HFpEF

	OR	95% CI	p	OR	%95 CI	p
Age	1,059	1.012-1.109	0.013	1.022	1.012-1.035	0.001
QRS duration	1.010	0.983-1.037	0.476			
LAD	1.302	1.138-1.490	0.001	1.220	1.110-1.350	0.002
P wave duratıon	1.651	1.120-1.235	0.005	1.33	1.240-1.550	0.03
Tpe	1.053	1.022-1.084	0.001	1.131	1.088-1.175	0.000
Selvester QRS score	2.446	1.783-3.555	0.001	1.588	1.352-1.755	0.000
Hs-CRP(mg/L)	1.655	1.256-2.122	0.000	1.436	1.115-1.848	0.005
NT-proBNP(pg/ml)	1.211	1.108-1.324	0.000	1.431	1.306-1.696	0.001
Hs-TnT(pg/L)	1.004	0.989-1.009	0.375			
LAVI	1.056	1.024-1.078	0.001	1.035	1.022-1.055	0.005

LAD: left atrium diameter; HFpEF: heart failure preserved ejection fraction; Tpe: Tpeak to end; LAVI: left atrial volume index; Hs-CRP: high sensitivity c-reactive protein; NT-proBNP: N-terminal B-type natriuretic peptide; Hs-TnT: high sensitive troponin T.

In the ROC analysis, the cut-off value of the S-QRS score was 5.5, with a sensitivity of 80.8% and a specificity of 77.2% (AUC: 0.880) (Figure 1). According to Kaplan-Meier analysis, mortality was higher in the group with an S-QRS score of ≥ 5.5 than in the group with an S-QRS score of < 5.5.(Long-rank, p:0.00) (Figure 2).

**Figure 1:** – ROC curve analysis of Selvester QRS score. AUC: area under the curve; CI: confidence interval.

**Figure 2:** – Kaplan Meier analysis of Selvester QRS score. The cardiac event rate was significantly higher in the high score (HS) group (green line) than in the low score (LS) group (blue line).

## Discussion

Our study was the first to investigate the predictive value of the S-QRS score for mortality in HFpEF. The ROC analysis showed a sensitivity and specificity of 80.8% and 77.2%, respectively. At the end of this study, the S-QRS score was found to be an independent risk factor for long-term mortality in patients with HFpEF.

Similar to previous studies, age, Hs-CRP, NT-proBNP, LA diameter, LA volume index, and Tpe were independent risk factors for mortality in patients with HFpEF. In clinical studies, mortality from all causes, mortality due to cardiovascular disease, HF, and hospitalization, were observed much more frequently in the elderly than in the young.^16,17^ Tromp et al.^18^ determined that the mortality rate was 6.9-fold higher, and the hospitalization rate was 16.9-fold higher in those over 85 years of age than in those under 55.^18^

In many studies, Hs-CRP has been shown to be a very important prognostic indicator of mortality due to inflammation and fibrosis in the pathophysiology of HFpEF.^19,20^L. Koller et al.^21^ reported that mortality from all causes increased 1.2-fold and cardiovascular mortality increased 1.32-fold in patients with elevated Hs-CRP, resulting from a mean follow-up of 9.7 years in 459 HFpEF patients.^21^ It has been reported that NT-proBNP released during increased myocardial wall stress due to a hypertrophic and small left ventricle, which is the characteristic feature of HFpEF, has predictive power for long-term morbidity and mortality in HFpEF patients, both in terms of basal levels and changes in these levels.^22^ Although the predictive value of natriuretic peptides is known to be lower in HFpEF patients than in HF low ejection fraction patients, they were shown to have the same predictive value in both HF groups in the study by van Veldhuisen et al.^23^

Tpe is an ECG marker that has received much attention in recent years. In many studies, transmural dispersion has been accepted as an indicator of repolarization abnormalities and has been shown to be associated with ventricular arrhythmias and sudden death.^24^Studies in HFpEF patients have concluded that Tpe is an important prognostic marker, proportional to disease severity and an independent risk factor for mortality.^25^

Measurement of the LA is a simple, reproducible, and commonly used parameter in clinical practice and research. In studies performed in HFpEF patients, increased LA diameter and volume index are considered prognostic indicators for many complications, such as atrial fibrillation, pulmonary hypertension, and cardiovascular mortality.^26^Rossi et al.^27^ pointed out in their prospective study that increased LA diameter increased mortality by 1.72-fold.^27^ The study by Pate et al. found that mortality increased by 0.9% with each millimeter increase in the LA volume index.^28^

The S-QRS score provides information about the size and location of myocardial scars by examining the QRS morphologic changes that occur due to ventricular depolarization changes resulting from myocardial fibrosis.^29^ Many autopsies and cardiac magnetic resonance imaging (MRI) studies have found a high degree of correlation between the S-QRS score and scar size.^30-32^ The prospective study by Liu et al.^33^ showed that cardiovascular mortality increased 1.46-fold in patients with elevated S-QRS scores. This resulted from a 2-year follow-up of 289 patients after MI with ST elevation compared to patients without it. In the study by Bignoti et al.,^13^ 228 patients who underwent transcatheter valve replacement for aortic valve stenosis were followed up for 36.2 ± 21.2 months and showed a 1.59-fold higher cardiovascular mortality rate in patients with high S-QRS scores.^13^ In the study by Hirawi et al.,^12^ a 1.32-fold higher rate of fatal cardiac events was observed in patients with high S-QRS scores after a mean follow-up of 4.5 ± 3.2 years in 91 patients with non-ischemic cardiomyopathy. In addition, a high correlation with the S-QRS score of collagen fraction measured by endomyocardial biopsy was found.^12^ Uyarel et al.^34^ demonstrated the development of a no-reflow phenomenon and high 30-day mortality after MI with ST elevation in patients with a high S-QRS score.^34^ In the study by Arisoy et al.,^35^ it was shown that a high S-QRS score is an independent risk factor for ventricular tachycardia and/or ventricular fibrillation in patients with non-ischemic cardiomyopathy.^35^ In the study by Chen et al.,^36^ which compared cardiac MRI and the S-QRS score in patients with hypertrophic cardiomyopathy, it was noted that the S-QRS score indicated the presence and size of the scar as accurately as cardiac MRI.^36^ Netsi et al.^37^ on the other hand, showed that the S-QRS score before implantation of cardiac resynchronization therapy (CRT) is one of the most important indicators of response to CRT treatment.^37^ Many studies have revealed that cardiac fibrosis is one of the most important pathophysiological mechanisms in patients with HFpEF. The autopsy study by Mohammed et al.,^38^ proved that epicardial coronary artery disease, microvascular infarcts, and gross and microscopic scars were more prevalent in HFpEF patients compared with the control group.^38^ In the cardiac MRI study by Garg et al.,^39^ fibrosis size was determined to be an independent risk indicator for mortality in patients with HFpEF.^39^ Cho et al.^40^ reported that fibrosis size was an independent risk factor for developing ventricular arrhythmias in HFpEF patients.^40^ In a cardiac MRI study by Kanagala et al.^41^ in patients with HFpEF, fibrosis size was found to be an independent risk factor for biventricular and LA remodeling, as well as hospitalization and mortality.^41^ The S-QRS score is a simple, inexpensive, and widely accepted scoring system that measures ventricular scar size and is obtained with a standard 12-lead ECG. In our study, the S-QRS score was found to be an independent risk factor for mortality in patients with HFpEF.

### Limitations

This study has many limitations. First, the number of patients included in the study is small, and the ECG records were not examined during routine examinations. In addition, values such as CRP and troponin, which are associated with subclinical myocardial damage, were not serially followed up. Cardiac MRI, the gold standard for measuring ventricular fibrosis, was not performed.

## Conclusion

The S-QRS score measured by standard 12-lead ECG was found to be an independent risk factor for mortality in patients with HFpEF. Therefore, it provides information on patient mortality even in the absence of cardiac MRI access and when other ECG parameters are normal. We recommend that the S-QRS score should not be neglected in evaluating high-risk patients.
